# Gestational Diabetes Mellitus and Future Cardiovascular Risk: An Update

**DOI:** 10.1155/2016/2070926

**Published:** 2016-11-13

**Authors:** S. Burlina, M. G. Dalfrà, N. C. Chilelli, A. Lapolla

**Affiliations:** Department of Medicine (DIMED), University of Padova, Via Giustiniani, No. 2, 35128 Padova, Italy

## Abstract

The prevalence of gestational diabetes mellitus is increasing in parallel with the rising prevalence of type 2 diabetes and obesity around the world. Current evidence strongly suggests that women who have had gestational diabetes mellitus are at greater risk of cardiovascular disease later in life. Given the growing prevalence of gestational diabetes mellitus, it is important to identify appropriate reliable markers of cardiovascular disease and specific treatment strategies capable of containing obesity, diabetes, and metabolic syndrome in order to reduce the burden of cardiovascular disease in the women affected.

## 1. Introduction

Gestational diabetes mellitus (GDM) is defined as any degree of glucose intolerance developing or first recognized during pregnancy that is not clearly overt diabetes. It affects from 5-6% to 15–20% of pregnancies worldwide, depending on population demographics, screening methods, diagnostic criteria in use, and maternal lifestyle [1]. The pathophysiological mechanisms behind the onset of GDM are still not well understood. In the second and third trimesters of pregnancy, there is a physiological increase of insulin resistance as a result of placental hormones such as estrogen, progesterone, human placental lactogen, human placenta growth hormone, and cortisol that antagonize the action of insulin [2]. The gradual decline in insulin sensitivity is considered a physiological mechanism that helps to provide glucose to the fetus, and it coincides with a gradual increase in the secretion of insulin to maintain normal glucose tolerance [3, 4]. Pregnancy is* per se* a hyperinsulinemic condition and GDM may develop if insulin secretion by the beta cells is unable to compensate the pregnancy-associated insulin resistance [5]. Most women with GDM are overweight or obese and have all the features of metabolic syndrome, but lean women with none of the common risk factors can develop GDM too.

Women with GDM are at greater risk of metabolic syndrome (characterized by central obesity, dyslipidemia, and insulin resistance) and type 2 diabetes years after their pregnancy [6, 7]. GDM progresses to type 2 diabetes in the years after pregnancy with a cumulative incidence in the range of 2.6–70%, from 6 weeks to 28 years postpartum [7].

Women who develop GDM are also at higher risk of overt cardiovascular disease (CVD) later in life [8]. While a diagnosis of type 2 diabetes in these women markedly raises their CVD risk [8], some studies have demonstrated that a diagnosis of GDM alone contributes to this risk, with or without any subsequent type 2 diabetes. In a cross-sectional study, 332 women with a history of GDM had a higher prevalence of CVD 29.9 years after the index pregnancy (adjusted OR: 1.85; 95% CI: 1.21–2.82), irrespective of any type 2 diabetes (OR: 1.56; 95% CI: 1.002–2.43) [8]. Retnakaran and Shah investigated the possible relationship between mild glucose tolerance in pregnancy and CVD risk in later life in a retrospective population-based cohort study [9]. They studied 13,888 women who developed GDM, 71,831 women who had an abnormal 50 g glucose test result but no GDM, and 349,977 women who had a normal response to the 50 g glucose challenge, with a median follow-up of 12.3 years. Compared with the women with normal glucose tolerance, the authors found an adjusted hazard ratio for CVD (acute myocardial infarction, coronary bypass, coronary angioplasty, stroke, and carotid endarterectomy) of 1.66 (95% CI: 1.30–2.13) for the GDM women and 1.19 (95% CI: 1.02–1.39) for the women with an abnormal glucose test result. Adjusting for the subsequent onset of type 2 diabetes led to attenuation of the hazard ratios for CVD, which became 1.25 (95% CI: 0.96–1.62) for the GDM group and 1.16 (95% CI: 0.99–1.36) for the group with an abnormal glucose test result. The authors concluded that even women with mild hyperglycemia in pregnancy, but no GDM, are at higher risk of subsequent adverse cardiovascular outcomes. It should be emphasized, however, that a large proportion of the elevated CVD risk in the abovementioned study could relate to the subsequent onset of type 2 diabetes, as suggested by the hazard ratios adjusted for this diagnosis.

Be that as it may, the effect of GDM on the risk of CVD remains to be fully elucidated: it is still not clear whether the association existing between GDM and CVD is independent of the increased risk of CVD associated with type 2 diabetes.

**Figure 1:**
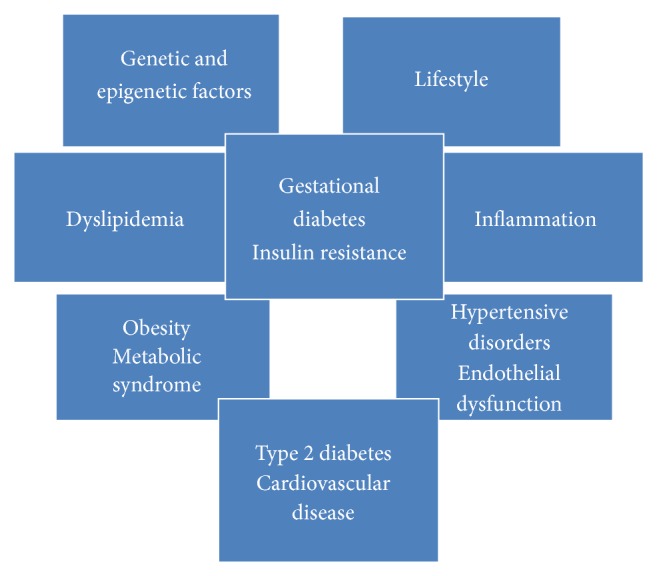
Relationship between GDM and subsequent cardiovascular disease: modifiable and unmodifiable risk factors.

In this paper, we review the relationship between common CVD risk factors and a history of GDM and take a look at potential new markers of CVD in such women (Figure 1).

## 2. Methods

A review of the international literature was conducted as regards the cardiovascular risk for women with GDM or a history of GDM. The keywords used were as follows: gestational diabetes mellitus, cardiovascular disease, cardiovascular risk, vascular disease, pregnancy, and pregnancy complication. Only data deriving from human studies and produced from 2005 onwards were considered in order to ensure that the evidence was topical. Literature dating from before 2005 was only included if it was particularly relevant. Data regarding patients with prior diabetes (type 1 or type 2) were not considered.

## 3. Common CVD Risk Factors in Women with a History of GDM

### 3.1. Hypertension

In the literature, there is plenty of evidence of a greater risk of hypertension in women with a history of GDM. Carr et al. [8] demonstrated that women with prior GDM were more likely to develop hypertension than women with no history of GDM (46.8% versus 37%; *p* < 0.001) and that any hypertension would be diagnosed at an earlier age in the former than in the latter (40 ± 1.0 versus 47.8 ± 0.9 years; *p* < 0.001). Kaul et al. [16] studied a large cohort of 240,083 women giving birth over a 10-year period. During this time, 14.9% of the nonobese women with a history of GDM developed hypertension; the hazard ratio, adjusted for maternal age, preeclampsia, parity, smoking status, ethnicity, and socioeconomic status, was 2.0 (1.8–2.2); and in the obese women with a history of GDM, the rate of hypertension rose to 26.8% and the hazard ratio to 3.7 (3.2–4.3). Goueslard et al. [17] recently reported on a large nationwide population-based retrospective study conducted in France, with a follow-up of 7 years. They considered 62,958 women with and 1,452,429 women without a history of GDM. The results of logistic regression analysis adjusted for age showed that GDM was associated with a significantly higher risk of hypertension, with an adjusted OR of 2.92 (2.77–3.08).

### 3.2. Dyslipidemia

Numerous published reports demonstrate that women with a history of GDM are more dyslipidemic. Like the situation seen for hypertension, Carr et al. showed that women who developed GDM were more likely to report a history of acquired dyslipidemia (33.9% versus 26.3%; *p* < 0.05), to take medication for dyslipidemia (18.4% versus 13.7%; *p* < 0.05), and to be diagnosed with dyslipidemia at a younger age (47.6 ± 1.3 versus 51.9 ± 1.0 years; *p* = 0.01) than women with no history of GDM [8]. In another study [18], women with singleton pregnancies who had GDM or normal glucose tolerance were examined from 2 to 24 months after their pregnancy: those with a history of GDM had higher total cholesterol (5.06 versus 4.56 mmol/L; *p* = 0.001), LDL-cholesterol (3.17 versus 2.57 mmol/L; *p* = 0.001), and triglyceride levels (1.02 versus 0.86 mmol/L; *p* = 0.01) and lower HDL-cholesterol levels (1.53 versus 1.73 mmol/L; *p* = 0.001). In a similar study population, Retnakaran et al. found GDM to be an independent predictor of total cholesterol, LDL-cholesterol, and triglyceride levels measured 3 months after delivery. These authors also demonstrated a stronger correlation between the area under the curve on the antepartum oral glucose tolerance test and postpartum levels of LDL-cholesterol and triglycerides, total cholesterol to HDL ratio, apoB, and apoB to apoA1 ratio (all *r* > 0.21; *p* < 0.0001) and an inverse relationship with HDL-cholesterol (*r* = −0.21; *p* < 0.0001), after adjusting for age, ethnicity, and family history of diabetes [19].

### 3.3. Metabolic Syndrome (Table 1)

Metabolic syndrome is characterized by abdominal obesity, hypertension, dyslipidemia, and abnormal glucose tolerance [20]. The condition carries a six- to eightfold higher risk of CVD and a two- to threefold higher CVD-related mortality rate by comparison with healthy controls [21].

Women who have had GDM are at high risk of developing metabolic syndrome. In a cohort of Caucasian women, for instance, the prevalence of metabolic syndrome 16 months after delivery was 9% among the women with a history of GDM and only 1% among controls (*p* < 0.01), when NCEP, ATP III criteria were applied [13]. This prevalence rose from 9% to 14.5% for the former and from 1% to 2% for the latter when IDF criteria were adopted (*p* < 0.001) [22]. Other studies on cohorts of Caucasian women with a follow-up ranging from 5 to 11 years after delivery found that the prevalence of metabolic syndrome among the women with a history of GDM ranged from 11.1% to 43%, as opposed to 4.6–6.1% in a control population [10–12]. A recent hospital-based cohort study found that the risk of metabolic syndrome 2–6 years after delivery was 2.4 times higher in women with a history of GDM than in those with normal glucose tolerance in pregnancy. Multivariate analysis indicated that a history of GDM predicted the onset of metabolic syndrome with an OR of 2.83 [14]. Noctor et al. [15] recently examined the prevalence of metabolic syndrome in women with a history of GDM according to the new criteria for the diagnosis of this condition [23]. Their sample consisted of 265 women with a history of GDM at a mean of 2.6 years after the index pregnancy and 378 women with normal glucose tolerance in pregnancy at a mean of 3.3 years after pregnancy. According to the ATP III criteria, 25.3% of the GDM women had metabolic syndrome as opposed to 6.6% of the controls. The authors also found that obesity confers a significant excess risk of metabolic syndrome in women who have had GDM, with an OR of 3.9 (95% CI: 2.0–7.9) for obese women with as opposed to without a history of GDM.

## 4. Early Changes in Vascular Structure and Function in Women with a History of GDM

Even women with a history of GDM who have no common CV risk factors are at greater risk of CVD than those with normal glucose tolerance in pregnancy. GDM seems to have a significant impact on endothelial function and structure, triggering the first step towards the development of atherosclerosis.

Carotid artery intima-media thickness (cIMT) is a subclinical measure of early atherosclerosis that strongly predicts heart disease and stroke, particularly in women [24]. In recent years, numerous studies have been published on cIMT in women who have had GDM. Bo et al. measured cIMT six and a half years after delivery in 82 women with and 113 without a history of GDM [25]. They found cIMT to be significantly higher in the former than in the latter, even among women with no components of metabolic syndrome, and irrespective of their BMI. cIMT was also significantly associated with a history of GDM in a multiple regression analysis, after adjusting for waist circumference, BMI, blood pressure, and blood glucose levels. Volpe et al. investigated cIMT two years after delivery in 28 women with and 24 without a history of GDM [26]. There were no differences between the two groups in terms of BMI, but the cIMT values were higher in the GDM women, though they were still within the upper limit of normal (0.57 ± 0.058 versus 0.51 ± 0.051 mm, *p* < 0.01). It is important to mention, however, that these groups also differed in terms of the principal components of metabolic syndrome (waist circumference, blood pressure, fasting plasma glucose, and triglycerides), which were all significantly higher in the GDM women than in the controls. In a population-based, multicenter, longitudinal, and observational study conducted by Gunderson et al. [27], 898 women with no diabetes or heart disease at the baseline subsequently had >1 delivery and then reported their GDM history and underwent cIMT measurement 20 years later. Among the women who developed no type 2 diabetes or metabolic syndrome during the 20-year follow-up, the mean cIMT was 0.023 mm greater for the women with a history of GDM in a model adjusted for age, race, parity, and prepregnancy BMI. On the other hand, the mean cIMT did not differ by GDM history among the women who developed type 2 diabetes or metabolic syndrome during the follow-up. The authors concluded that a history of GDM can be considered a risk factor for atherosclerosis even before the onset of diabetes or metabolic syndrome.

Another proposed surrogate marker for the early detection of atherosclerosis is the flow-mediated dilation (FMD) of the brachial artery [28], which is an indicator of endothelial dysfunction—one of the earliest signs of atherosclerosis [29]. Anastasiou et al. measured FMD 3–6 months after delivery in nonobese and obese women with a history of GDM [30]. They found FMD to be significantly lower in both nonobese and obese GDM women than in control women. They also showed that FMD correlated inversely with BMI, serum total cholesterol, and basal insulin resistance (assessed with a homeostasis model). Davenport et al. found FMD to be impaired in GDM women already 7–9 weeks after delivery. In this particular study, a sample of women was divided into 4 groups: those with a history of GDM who had become normoglycemic; those with a history of GDM who remained hyperglycemic; those with no history of GDM; and those who had never been pregnant. FMD was significantly lower in the former two groups than in the latter two. Interestingly, FMD no longer differed significantly between the four groups after controlling for glucose AUC, which goes to show the importance of postpartum hyperglycemia in determining endothelial dysfunction after pregnancy [31]. After adjusting for age and blood pressure levels, Fakhrzadeh et al. reported a significant reduction in FMD 4 years after delivery in women with a history of GDM [32] by comparison with control women (26 ± 0.11% versus 19.32 ± 0.05%; *p* = 0.003). They also reported finding no correlation between FMD and inflammatory parameters, lipid profile, or insulin resistance indices; they did not consider glucose AUC.

Hannemann et al., on the other hand, found no differences in FMD between women who had experienced GDM five years earlier and control women matched for age, BMI, and smoking habits [33]. Brewster et al. likewise found no differences in FMD between women with a history of GDM and control women 6 years after delivery (mean 8.5% versus 9.3%, *p* = 0.61) [34]. There is therefore no way of saying for sure that FMD is impaired in later years in women who have had GDM. It is worth noting that most of the studies that did find a worse FMD were conducted soon after delivery, so it may be that this impairment is an early vascular function abnormality that may return to normal with time if glucose tolerance returns to normal; that is, FMD could be influenced mainly by hyperglycemia. Supporting this hypothesis, two studies have demonstrated that FMD is reduced during pregnancy in women with GDM. Paradisi et al. found FMD to be significantly lower in GDM women than in controls (4.1 ± 0.9% versus 10.9 ± 1.1%; *p* < 0.0001) in the third trimester of pregnancy [35]. They found too that glucose AUC independently influenced FMD (*p* < 0.0001). In another cross-sectional study on pregnant women with GDM (n. 19) or preeclampsia (n. 42) and controls with normal glucose tolerance and blood pressure (n. 19), Guimarães et al. also demonstrated a significantly reduced FMD in the women with GDM or preeclampsia by comparison with the controls, and they suggested the possibility of endothelial injury in such patients [36].

In this setting, Caliskan et al. recently studied the coronary flow velocity reserve (CFVR), which reflects coronary microvascular function, in women with a history of GDM 6 months after delivery. They found this parameter to be significantly reduced in the GDM women by comparison with controls whose glucose tolerance remained normal in pregnancy (2.34 ± 0.39 versus 2.83 ± 0.21; *p* < 0.001) and also that insulin resistance, hyperglycemia, and oxidative stress markers were negatively associated with CVFR. On multivariate analysis, the authors also found an independent association between CFVR and GDM (*p* = 0.02) [37].

## 5. New Markers

Endothelial dysfunction is believed to be an important initiating factor in the development of atherosclerosis [29]. Like circulating levels of systemic inflammatory markers, the levels of some adipokines have also been associated with endothelial dysfunction and atherosclerosis. Apelin, a recently discovered adipocytokine, is an endogenous ligand of the G protein-coupled receptor APJ [38] that is produced by adipose tissue and expressed in various tissues (brain, lung, heart, pancreas, kidney, and endothelial cells) and believed to have a role in the cardiovascular system [39].

In a recent study, 141 women with a history of GDM and 49 age- and BMI-matched healthy control women were tested for circulating apelin, IL-6, and plasminogen activator inhibitor levels and IMT and took an oral glucose tolerance challenge. The results showed that plasma apelin levels were lower in women with a history of GDM and, in multiple regression analysis, they were negatively associated with fasting and postload glucose, IL-6, and carotid IMT. Suppressed apelin levels are therefore associated with a higher cardiovascular risk in women with a history of GDM [40].

Subclinical inflammation is another major risk factor for future CVD in the general population, and the higher risk of CVD later in life for women with a history of GDM is potentially at least partly due to inflammatory mechanisms [13]. Although several studies have demonstrated higher levels of markers reflecting vascular inflammation in women who have had GDM, the mechanisms behind vascular injury and CVD are not well understood [13].

Osteoprotegerin (OPG) is a soluble member of the tumor necrosis factor (TNF) receptor superfamily that inhibits osteoclast maturation and protects bone from normal osteoclast remodeling [41]. OPG has an important role in lymphocyte development and apoptosis too, and its levels have been associated with CVD [42]. In a cross-sectional case-control study, 128 women with a history of GDM and 67 age-matched controls were considered for a diagnosis of metabolic syndrome according to the criteria of the American Heart Association (AHA), and their glucose and insulin levels, serum lipids, OPG, and cIMT were also measured. The women who were confirmed to have metabolic syndrome had higher OPG levels than those who were not, or healthy controls; and serum OPG levels were found to be associated with obesity, insulin resistance, and cIMT [43].

Pentraxin 3 (PTX3) is an essential component of innate immunity induced by various inflammatory stimuli. It is produced by endothelial cell macrophages and granulocytes at sites of inflammation [44] and may have a cardioprotective role: higher levels in patients with CVD reflect a beneficial response in terms of reduced immune activation [45].

Lekva et al. considered oral glucose tolerance test findings, lipid profiles, PTX3 levels, and arterial stiffness in 300 women during pregnancy and 5 years afterwards. Early in pregnancy and 5 years later, PTX3 levels were lower in the women who developed GDM, and they were associated with BMI. Low PTX3 levels in early pregnancy were inversely correlated with metabolic risk factors for CVD (such as body composition, arterial stiffness, dyslipidemia, and a history of GDM) 5 years after delivery. Low plasma concentrations of PTX3 in early pregnancy are therefore associated with the subsequent onset of GDM and a higher risk of CVD later on [46].

## 6. Conclusions

In conclusion, numerous studies have demonstrated an increased risk of type 2 diabetes, metabolic syndrome, and CVD after pregnancy in women who develop GDM, but the mechanisms contributing to the vascular dysfunction seen in GDM women remain uncertain. For the time being, no validated markers of this vascular risk are identifiable before the onset of diabetes, metabolic syndrome, or cardiovascular morbidity. Novel potential early markers have recently been proposed, but further investigations on larger samples and longitudinal studies are needed to confirm their value. Given the rising prevalence of GDM, future studies should aim to identify strong early markers of CVD in women who develop this condition, and specific strategies are warranted to prevent or reduce obesity, diabetes, metabolic syndrome, and consequent CVD, in this particular population.

## Figures and Tables

**Table 1:** Frequency of metabolic syndromes in women with a history of gestational diabetes mellitus, according to the literature.

Authors	Follow-up	Prevalence of metabolic syndrome (%)	Diagnostic criteria for metabolic syndrome
Bo et al., 2004 [10]	8.5 yrs	21	ATP III
Albareda et al., 2005 [11]	5 yrs	11.1	ATP III
Lauenborg et al., 2005 [12]	9.8 yrs	38.4	WHO
Di Cianni et al., 2007 [13]	16 months	9	ATP III
Vilmi-Kerälä et al., 2015 [14]	2–6 yrs	23.1	ATP III
Noctor et al., 2015 [15]	2.6 yrs	25.3	WHO
